# Mother–child interactions in adolescents with borderline personality disorder traits and the impact of early life maltreatment

**DOI:** 10.1186/s13034-023-00645-4

**Published:** 2023-08-10

**Authors:** Katharina Williams, Leonie Fleck, Anna Fuchs, Julian Koenig, Michael Kaess

**Affiliations:** 1 Department of Child and Adolescent Psychiatry, Centre for Psychosocial Medicine, Medical Faculty, University of Heidelberg, Blumenstr. 8, Heidelberg, Germany; 2 Institute of Psychology, University of Heidelberg, Heidelberg, Germany; 3 Faculty of Medicine and University Hospital Cologne, Department of Child and Adolescent Psychiatry, Psychosomatics and Psychotherapy, University of Cologne, Cologne, Germany; 4 University Hospital of Child and Adolescent Psychiatry and Psychotherapy, University of Bern, Bern, Switzerland

**Keywords:** Borderline personality disorder, Mother–child interaction, Interpersonal dysfunction, Early life maltreatment, BPD specific therapies

## Abstract

**Background:**

Early detection and intervention of borderline personality disorder (BPD) in adolescence has become a public health priority. Theoretical models emphasize the role of social interactions and transgenerational mechanisms in the development of the disorder suggesting a closer look at caregiver-child relationships.

**Methods:**

The current study investigated mother-adolescent interactions and their association with adolescent BPD traits by using a case–control design. Thirty-eight adolescent patients with ≥ 3 BPD traits and their mothers (BPD-G) were investigated in contrast to 35 healthy control dyads (HC-G). Maternal, adolescent and dyadic behavior was coded using the Coding Interactive Behavior Manual (CIB) during two interactions: a fun day planning and a stress paradigm. Additional effects of maternal and/or adolescent early life maltreatment (ELM) on behavior were also explored.

**Results:**

BPD-G displayed a significantly lower quality of maternal, adolescent and dyadic behavior than the HC-G during both interactions. Maternal and adolescent behavior was predicted by BPD traits alone, whilst dyadic behavior was also influenced by general adolescent psychopathology. Exploratory analyses of CIB subscales showed that whilst HC-G increased their reciprocal behavior during stress compared to the fun day planning, BPD-G dyads decreased it. Maternal ELM did not differ between groups or have any effect on behavior. Adolescent ELM was correlated with behavioral outcome variables, but did not explain behavioral outcomes above and beyond the effect of clinical status.

**Discussion/Conclusion:**

Our data suggest a stronger focus on parent–child interactions in BPD-specific therapies to enhance long-term treatment outcomes in adolescent BPD patients. Further research employing study designs that allow the analyses of bidirectional transactions (e.g. longitudinal design, behavioral microcoding) is needed.

## Introduction

Borderline personality disorder (BPD) is a complex mental disorder characterized by severe impairments in interpersonal functioning, instability of self and emotion dysregulation [3]. It is associated with high suicide rates, serious self-harm, long-term occupational disabilities and poor physical health [2, 15, 47].

Although the diagnosis of BPD in adolescence is still controversially discussed in clinical practice [19], researchers in the field [21, 22, 94] as well as current international guidelines for BPD that are based on empirical data [4, 67] strongly suggest diagnosing BPD and subthreshold BPD patterns in adolescence, a particularly sensitive period for the onset of mental disorders. An early diagnosis of BPD seems to be beneficial to the course of treatment [2, 13, 42, 49], adolescents receiving BPD specific therapy benefit from a short-term reduction of BPD symptoms and enhanced personality and psychosocial functioning [2, 21, 79, 95]. In order to improve psychiatric and psychotherapeutic care and therefore treatment outcomes for BPD patients, a better understanding of underlying processes in adolescence distinguishing healthy from pathological development is necessary [90]. Developmental theories of BPD [36, 57, 81] emphasize the role of interpersonal relationships, specifically the caregiver-child relationship, in the etiology, expression and maintenance of BPD.

### Interpersonal functioning and BPD

Although remission rates for BPD are promising with an average of 60% in the course of 5–15 years [2], individuals with BPD often continue to suffer from severe interpersonal impairments [42, 58, 83, 90]. The cumulation of stressful interpersonal life events can further aggravate poor psychosocial functioning in adult BPD patients [68]. Also, acute BPD symptoms like self-harm behavior, intense anger or depressive symptoms often occur in interpersonal contexts [44, 66, 69, 71] and the perceived quality of interpersonal relationships influences current BPD symptomatology and vice versa [44]. Interpersonal experiences and processes therefore seem to influence present BPD symptoms but also the long-term psychosocial development of BPD individuals. As early relationship experiences form our later expectation of and behavior in social interactions [1, 37, 40], a closer look at caregiver-child relationships and their dyadic interaction is warranted.

Many adolescent BPD patients still live at home and experience maladaptive relationships with their caregivers [46]. This becomes especially relevant when considering the role of transgenerational transmission of mental disorder and trauma in the development of the disorder (see [48]. Several therapeutic approaches for adolescent BPD (e.g. [73]: dialectic-behavioral therapy, DBT, [76]: mentalization-based treatment, MBT, [35]: adolescent identity training, AIT) have already addressed this fact by including caregivers in the treatment of the disorder. However, although first evidence from these treatments suggests an enhancement of psychosocial functioning for adolescent BPD patients (e.g. [79], they still seem to profit less from these therapies than adult BPD patients [86]. More research is needed in order to understand familial interactional patterns, detect risk and protective factors and to identify potential windows for interventions.

### Caregiver-child interactions and BPD

According to Linehan’s Biosocial Theory (1993; see also [23], BPD develops as a result of child vulnerability (e.g. impulsivity, emotional sensitivity) interacting with an invalidating social environment. Important social risk factors include parental psychopathology, poor quality of the parent–child relationship, dysfunctional parenting practices and early life maltreatment [8, 15, 84].

Past research has mainly focused on the influence of parental caregiving, i.e. parent-driven effects, on the development of BPD pathology. In community samples, maladaptive parenting (e.g. chaotic parenting, physical maltreatment) was identified as predictive of adolescent BPD symptoms [5, 8]; validating parenting (emotional support, involvement), on the other hand, could have a protective effect on developing BPD symptoms [39]. Also in clinical samples, adolescents and young adults with BPD report that their parents displayed several problematic parenting practices (e.g. emotional withdrawal, parental inconsistencies, invalidation of thoughts and feelings; [7, 10, 14, 46, 63, 80, 89, 91]. Studies investigating child-driven effects have focused on temperament-related features: a review of Boucher et al. [14] summarized that parents of children with BPD often describe their child as “unusually sensitive” or with a “difficult temperament” early on.

The studies reviewed above relied on the retrospective reports of BPD patients and/or their parents by applying self-report questionnaires and interviews. Self-reported experiences, however, may be influenced by recall bias and at least in parts by the BPD symptomatology itself [18, 30]. The observation and professional coding of behavior during caregiver-child interactions offers a chance to address this problem. Regarding parent-driven effects, longitudinal studies using high-risk community samples showed that maternal withdrawal and hostility displayed during parent-toddler interactions predicted BPD symptoms in early adulthood [18, 61]. Maternal insensitivity during mother–child interactions at infancy, preschool and adolescence was associated with adolescent BPD traits [17]. On the child’s side, disorganized-controlling behavior at age 8 was predictive of early adult BPD symptoms [61].

Surprisingly few studies have observed (and reported) parent and child behavior at the same time in the context of current BPD symptomatology. It has also been suggested to observe behavior not only on an individual but also on a dyadic level, as mother and child are most likely influencing each other during interactions [92]. In at-risk community samples with adolescents, dyadic negative escalation and disoriented/role-confused behavior during a conflict discussion between mothers and their adolescent children were associated with more BPD traits [60, 92]. Positive dyadic behavior, however, was related to decreases in adolescent girls’ BPD severity scores over time [92]. Dixon-Gordon et al. [28] identified adolescent negative affect during interaction as a possible risk factor for BPD traits but only when mothers showed low support/validation and high problem solving during a conflict discussion task. In a clinical sample comparing young adults with BPD with healthy controls, BPD patients and their mothers displayed more disorganized behavior during a conflict discussion task [54]. Whilst overall collaboration as a marker for dyadic behavior during conflict discussions did not predict adolescent BPD traits in a high-risk community sample [60], it contributed to a more secure attachment profile in the clinical sample of young adults [54]. Fleck et al. [34] observed interactions in two community samples. At age 9, they found less maternal structuring and more child withdrawal during a conflict discussion task to be associated with BPD traits, but no relations during a fun day planning task. At age 14, less maternal sensitivity and structuring, more maternal intrusiveness and less child engagement during the conflict discussion task and more child withdrawal during the fun day planning was related to BPD traits. Associations between dyadic behavior and BPD traits in adolescents were significant during the conflict discussion task, but not during the fun day planning.

In summary, parental and child behavior in caregiver-child transactions seem to be altered and more conflict-driven when child or adolescent BPD traits are present. This seems to be especially relevant in stressful contexts (e.g. [34], when problems with emotion regulation would become noticeable. There is also first evidence that positive parental and dyadic behavior might mitigate the development of the disorder [39, 92]. Although many studies have identified specific maladaptive parenting practices that seem to foster the development of BPD, parental behavior was rarely investigated during actual mother–child interactions. Studies that have observed interactional behavior mainly focused on either parent- or child-driven effects in community samples.

To the best of our knowledge, only four studies have studied the influence of current BPD traits on parent, child and dyadic behavior during the same interaction [34, 54, 60, 92]; the only study including a case–control design focused on young adults rather than adolescents [54]. Only one study compared observed mother–child interactions in a positive versus a stressful context [34], although context seems to have an impact on relations between mother–child interactions and child behavioral problems [27].

The present study addresses these limitations by comparing a clinical adolescent sample with BPD symptoms and their mothers with healthy control dyads, observing maternal, adolescent and dyadic behavior during a positive interaction and a stress paradigm.

### Early life maltreatment, BPD and parent–child interactions

An overwhelming body of literature has identified ELM as a contributing factor to the development of BPD traits in children, adolescents and adults [16, 45, 70, 94]. Lyons-Ruth et al. [61] suggested that the assessment of parent–child interactions should include ELM as a possible influential factor. Previous research has focused mainly on maternal ELM and its influence on maternal behavior in mother–child interactions: mothers who have experienced ELM are more likely to show maladaptive parenting, including parental behavior that was previously associated with the development of BPD, such as psychological control, maternal hostility or harsh punishment [77, 88].

Although some of the above-described studies have reported child or adolescent ELM and have shown associations with the development of BPD traits (e.g. [18], the influence of child ELM on behavior in mother–child interactions was rarely considered. Maternal withdrawal during a mother-toddler interaction, child disorganized-controlling behavior at age 8 and less collaborative, more mutual punitive and disoriented/out-of-context behavior during discussions between young adults and their parents seem to be influenced by the severity of the trauma the child/adolescent has experienced in the past [60, 61]. Further research is needed to disentangle the effects of maternal/adolescent ELM and BPD pathology on caregiver-child interactions and how this may facilitate the development of BPD.

### The aim of the present study

With the present study, we aimed at expanding prior research on the observation of caregiver-child transactions by using a case–control group design. Specifically, we wanted to know how a clinical group of adolescents with BPD traits (BPD-G) differs from a healthy control group (HC-G) in maternal, adolescent and dyadic behavior during mother-adolescent interactions in a positive versus a stress context. Additionally, we wanted to investigate the role of maternal and adolescent ELM and how it might independently or additionally to adolescent BPD symptomatology contribute to behavior in this context.

Firstly, and consistent with prior literature, we expected maternal, adolescent and dyadic behavior to be of less quality in the BPD-G than in the HC-G. During stress, we assumed this group difference to be larger than during the fun day planning, i.e. the BPD-G was expected to show more dysfunctional behavior during stress -when emotion regulation difficulties might come into play- than during fun day whilst we did not expect the HC-G to significantly change behavior between contexts. We were also interested in exploratory analyses on subscale level to identify specific behavior (e.g. maternal intrusiveness vs. maternal sensitivity), that might explain differences between groups and/or contexts.

Secondly, we assumed that BPD-G mothers and adolescents experienced more ELM than subjects in the HC-G. As a large body of research suggests (see [88], we expected maternal ELM to influence maternal and dyadic behavior in both interactions; no specific assumptions were made for adolescent behavior. Regarding the influence of adolescent ELM on behavior we expected all behavioral outcomes to be affected, following indications of prior research [60, 61]. Lastly, we explored whether adolescent ELM has an effect on interactional behavior above and beyond the effect of group membership.

## Method

### Recruitment and participants

A-priori power analyses for group comparisons utilizing an anticipated effect size of *d* = 0.8, a desired statistical power level of 80% and a probability level of α = 0.05 revealed a minimum sample size of *n* = 26 per group. We therefore aimed at recruiting a total of 30 clinical dyads (BPD-G) and 30 healthy control dyads (HC-G). Recruitment took place from 06/2018 to 01/2021. BPD-G adolescents were recruited in our outpatient clinic for risk-taking and self-harm behavior (AtR!Sk; [50, 51]. Patients had to meet ≥ 3 criteria of BPD, which was assessed by trained clinical psychologists using the Structured Clinical Interview for DSM-5-Personality Disorders (SCID-5-PD; [33]. BPD-adolescents were also screened for other psychiatric disorders using the Mini International Neuropsychiatric Interview for Children and Adolescents (MINI-KID; [82]. HC-G were recruited via advertising and the local residents’ registration office and matched to BPD-G according to adolescent sex and age, and adolescent and maternal education. HC-G dyads were excluded if adolescents fulfilled criteria for any current or lifetime disorder (assessed with the MINI-KID or if mothers had received any psychotherapeutic/psychiatric treatment in the 2 years prior to the study. HC-G adolescents were also screened for BPD traits using the SCID-5-PD [33]. Further exclusion criteria for all mothers and adolescents were a diagnosis of schizophrenia and/or autism. As the study included biological measures (not reported in this manuscript), exclusion criteria for both groups were serious somatic illness, neurological disorder or cardiac/hypothalamus–pituitary–adrenal system dysfunction. Also, mothers had to be the primary caregiver.

From a pool of 353 possible participants (BPD-G = 161, HC-G = 192), 294 (83.3%) were contacted and screened for exclusion criteria (reasons for not being contacted: no phone number/email available, BPD-G: still waiting for or in the process of the clinical assessment of AtR!Sk, HC-G: no match for BPD-G). 205 of the contacted dyads [BPD-G: 102(63.4%); HC-G: 103(53.6%)] could not be included due to lack of interest (BPD-G: 51, HC-G: 46), somatic illness (BPD-G: 12; HC-G: 17), lack of time (BPD-G: 11; HC-G: 10), being too young or too old (BPD-G: 11; HC-G: 5), mother not being the primary caregiver or not being available (BPD-G: 9), no match for BPD-G (HC-G: 5), insufficient language skills (BPD-G: 4; HC-G: 2), psychiatric illness according to exclusion criteria (BPD-G: 2; HC-G: 12) or giving wrong contact information (BPD-G: 2; HC-G: 6). From 89 (25.2%) included dyads, 16 (18%) became dropouts during the course of the study: One BPD-G turned 18, one BPD-G mother reported a somatic illness, 9 of HC-G adolescents reported psychopathology of any kind, and some dyads lost interest in the study in both groups (BPD-G: 3; HC-G: 2).

Finally, 38 adolescent patients between 12;0 and 17;0 years (*mean* = 15.6, *sd* = 1.13) and their mothers formed the BPD-G; 35 healthy dyads formed the HC-G (adolescents aged between 14;0 and 17;0, *mean* = 15.5, *sd* = 1.25). Adolescents were mostly female (BPD-G: 84.2%, HC-G: 80%) and on track for higher education. Mothers were well educated and the majority part-time or full-time employed. All participants were of European ancestry. For a detailed sample description see Table 1.

**Table 1:** Sociodemographic Characteristics of the Sample

	HC-G	BPD-G		
	(n = 35)	(n = 38)		
	mean(sd)	mean(sd)	t value	p value
Mothers				
Age	48.20 (5.43)	46.68 (6.15)	1.118	0.267
	n (%)	n (%)	χ^2^ value	p value
Highest degree			2.107	0.575^a^
Lower secondary school	3 (8.57%)	5 (13.2%)		
Intermediate secondary school	7 (20.0%)	12 (31.6%)		
University entrance diploma	8 (22.9%)	6 (15.8%)		
University degree	17 (48.6%)	15 (39.5%)		
Employment			2.949	0.400^a^
Full-time at home^1^	3 (8.57%)	6 (15.8%)		
Part-time employment	19 (54.3%)	18 (47.4%)		
Full-time employment	13 (37.1%)	12 (31.6%)		
Other^2^	0 (0.00%)	2 (5.26%)		
Adolescents	mean (sd)	mean (sd)	t value	p value
Age	15.49 (1.25)	15.61 (1.13)	− 0.429	0.670
	n (%)	n (%)	χ^2^ value	p value
Gender			0.027	0.870
female	28 (80.0%)	32 (84.2%)		
male	7 (20.0%)	6 (15.8%)		
School form			3.925	0.159^a^
Lower secondary school	1 (2.86%)	4 (10.5%)		
Intermediate secondary school	10 (28.6%)	16 (42.1%)		
University entrance diploma	24 (68.6%)	18 (47.4%)		

*HC-G* healthy control group, *BPD-G* borderline personality disorder group

^a^Fisher’s Exact Test

^1^e.g. housewife, retired, unemployed

^2^temporary leave of absence, work on minijob-basis

### Procedure

Dyads were invited to our laboratory in Heidelberg for two appointments (t1 and t2) over a 3 week period. At t1, clinical assessment (interviews and questionnaires) and a computer task were performed. At t2, two 10-min-long standardized mother-adolescent interactions (a positive interaction that was, after a resting period, followed by a stress task) were videotaped. During the positive interaction, dyads were asked to plan positive activities both individuals would benefit from and enjoy. The stress task was loosely based on the Parent–Child-Challenging Task (PCCT) by Lunkenheimer et al. [59] and has, to the best of our knowledge, not been used before. During the stress task, the adolescent was presented with a tangram. The dyad was told that other adolescents were able to solve the tangram without any issues, when in fact it was too difficult to work out the tangram in the allotted timeframe. Additionally, the examiner would carefully observe their approach and make notes about their performance. Mothers were instructed to support their child but not to solve the puzzle for them. After 5 min, it was stated that the child was unsuccessful in completing the task and therefore an easier tangram would be presented (which was actually even more difficult to solve than the first one).

Preliminary analyses confirmed that mothers and adolescents reported significantly more negative affect [mothers: *t*(72) = -5.731, *p* < 0.001; adolescents: *t*(72) = -6.706, *p* < 0.001] and less positive affect (mothers: *t*(72) = 2.66, *p* < 0.01; adolescents:* t*(72) = 3.15, *p* < 0.01) in the stress task in comparison to the positive interaction.

Before, during and/or after interactions, physiological data (functional near-infrared spectroscopy, electrocardiography, saliva sampling) was retrieved. The physiological data as well as the computer task were not analyzed in the present study and will therefore not be described further.

### Measures

#### Clinical assessment

Adolescents of both groups filled in the Strength and Difficulties Questionnaire (SDQ; [41] to self-assess emotional and behavioral problems (*α* = 0.89). Mothers’ psychopathology was screened in both groups with the Symptom Checklist 90-Revised (SCL-90-R; [25], German version by [38],* α* = 0.98). As our study focused on interpersonal behavior, we specifically investigated the SLC-90-R subscale *interpersonal sensitivity*. Additionally, we assessed attachment security of mothers with the Vulnerable Attachment Style Questionnaire (VASQ; [11, 74] with higher values indicating higher attachment insecurity (*α* = 0.77).

#### Childhood maltreatment

The Childhood trauma questionnaire (CTQ, [9], German version by [29] was used to assess traumatic childhood events of adolescents and their mothers in self-report. In the present study, only total CTQ scores were used, with higher scores indicating a more severe history of childhood abuse and/or neglect (*α*_*mothers*_ = 0.94; *α*_*adolescents*_ = 0.92). The psychometric properties of the German version were found to be satisfying [78].

#### Quality of mother-adolescent interactions

Mother-adolescent interactions were rated using the Coding Interactive Behavior Manual (CIB) by Feldman [31]. 56 behavioral codes were rated from 1 (low) to 5 (high). These codes form maternal, child and dyadic scales: Maternal sensitivity (*α* = 0.89), maternal structuring (*α* = 0.78), maternal intrusiveness (*α* = 0.79), child engagement (α = 0.90), child compliance (*α* = 0.89), child withdrawal (α = 0.88), dyadic reciprocity (*α* = 0.91) and dyadic negativity (*α* = 0.83; for details about scale-item assignment see [34]. Additionally, total scales were built: the mother’s total score was calculated by subtracting maternal intrusiveness from sensitivity and structuring (*α* = 0.81), the child’s total score by subtracting child withdrawal from engagement and compliance (*α* = 0.92) and the dyadic total score by subtracting negativity from reciprocity (*α* = . 90). Therefore, higher total scores represent higher quality of behavior. Two raters were trained and certified by the instrument’s author, two additional trainers were trained and closely supervised by them. 41% of the videos were rated by at least two raters (interrater agreement 88%; Cohen’s Kappa = 0.77).

### Data analysis

All analyses were carried out with R (v1.4.1717; [72]. Per group, one stress task was missing due to malfunction of the video camera [*n* = 2 (2.74%)]. Welch’s t-tests were applied to calculate group differences for continuous variables [24], chi-square-tests for categorical variables.

For the analyses of the CIB data (BPD-G vs. HC-G and positive vs. stress context), robust two-way mixed Analyses of Variance (ANOVA) using 20% trimmed means were calculated via the *WRS2* package [64], as variance homogeneity could not be met. Although normal distribution could be assumed (sample sizes > 30), it was visually verified and tested with the Shapiro–Wilk-Test. Greenhouse–Geisser corrections were made when the assumption of sphericity was violated. In our main analyses, Bonferroni-Holm correction for multiple testing was applied across effects that were applying to the same hypothesis (i.e. for group differences, context differences and group x context interactions we controlled for three comparisons per hypothesis).

To ensure that the group effect on behavior is not an effect of general psychopathology but specific to BPD traits, we transformed all values into z-values and then calculated Pearson correlation coefficients to determine the association between SDQ and the CIB scales. When significant correlations were found, we calculated regression analyses with group as a predictor and the respective CIB scale as an outcome variable, controlling for SDQ.

To determine how much additional variance in behavior could be explained by maternal and/or adolescent ELM, again all values were z-transformed and Pearson correlation coefficients were calculated. Significant correlations were further investigated in hierarchical linear regression models (step1: group as a single predictor, step2: group and CTQ as predictors).

When homoscedasticity could not be met in the above-described regression models, HC4-method for robust standard errors was applied.

## Results

### Psychopathology of adolescents and their mothers

In the BPD-G (*range* = 3–8 BPD criteria), 19 (50%) adolescents fulfilled the BPD diagnosis. 9 (23.7%) BPD-G adolescents were diagnosed with F10-F19 diagnoses, 29 (76.3%) with F30-F39 diagnoses, 16 (42.10%) with F40-F49 diagnoses and 8 (21.05%) with F90-F99 diagnoses. 30 (78.95%) patients fulfilled at least two or more diagnoses. Adolescents of the BPD-G reported significantly more emotional and behavioral problems in the SDQ than the adolescents of the HC-G (*p* < 0.001); BPD-G mothers reported significantly more psychopathology in the SCL-90-R (*p* = 0.001), significantly more interpersonal sensitivity in the respective SCL-90-R subscale (*p* = 0.033) and significantly more attachment insecurity in the VASQ (*p* = 0.028) than HC-G mothers (for details see Table 2). However, on additional exploratory analyses we did not find any significant correlations between maternal interpersonal sensitivity and attachment insecurity and maternal, adolescent or dyadic behavior (*r* = 0.00 to *r* = 0.14).

**Table 2:** Sample Psychopathology and Early Life Maltreatment

	HC-G	BPD-G			
	(*n* = 35)	(*n* = 38)			
	mean(sd)	mean(sd)	t value	p value	Cohen’s* d*
Adolescents					
BPD criteria	–	4.74 (1.41)	–	–	–
SDQ total	8.14 (3.84)	19.9 (5.98)	− 10.084	< 0.001^***^	2.322
CTQ total	28.7 (4.06)	41.8 (10.8)	− 6.931	< 0.001^***^	1.574
Mothers					
SCL-90-R	0.32 (0.26)	0.66 (0.54)	− 3.432	0.001^**^	0.784
Interpersonal sensitivity	0.35 (0.32)	0.61(0.65)	2.169	0.033^*^	0.508
VASQ	53.69 (6.94)	58.11 (9.57)	2.242	0.028^*^	0.525
CTQ total	37.7 (12.0)	40.5 (13.9)	− 0.918	0.362	0.214

*HC-G* healthy control group, *BPD-G* borderline personality disorder group, BPD borderline personality disorder, *SDQ* Strengths and Difficulties Questionnaire, *CTQ* Childhood Trauma Questionnaire, *SCL-90-R* Symptom Checklist 90-Revised, *VASQ* Vulnerable Attachment Style Questionnaire

^**^p < 0.01. ***p < 0.001

### Early life maltreatment

BPD-G adolescents reported a significantly higher CTQ total score in comparison to the HC-G (*p* < 0.001). Mothers’ CTQ, however, did not significantly differ between groups (*p* = 0.365). Details can be obtained from Table 2.

### Quality of mother-adolescent interactions

Means and standard deviations of CIB scores from both interactions can be obtained from Table 3. For our main analyses, robust two-way mixed-model ANOVAs revealed a significant group effect for maternal (*p*_*adj*_ = 0.003), adolescent (*p*_*adj*_ = 0.003) and dyadic behavior (*p*_*adj*_ = 0.003): the BPD-G showed a significantly lower quality of maternal, adolescent and dyadic behavior in both interactions than the HC-G. For context, no significant main effects were found. Group x context interactions were also not significant (details can be obtained from Table 4).

**Table 3:** Descriptives of Maternal, Adolescent and Dyadic Behavior during Fun Day Planning and a Stress Paradigm

	Fun day planning		Stress paradigm	
	HC-G	BPD-G	HC-G	BPD-G
	(*n* = 35)	(*n* = 38)	(*n* = 35)	(*n* = 38)
	mean(sd)	mean(sd)	mean(sd)	mean(sd)
Mothers: CIB total	2.33 (0.34)	2.05 (0.58)	2.24 (0.28)	1.98 (0.44)
Maternal sensitivity	3.57 (0.58)	3.29 (0.72)	3.28 (0.49)	2.86 (0.76)
Maternal intrusiveness	1.18 (0.22)	1.42 (0.63)	1.26 (0.37)	1.30 (0.38)
Maternal structuring	4.58 (0.41)	4.29 (0.66)	4.71 (0.33)	4.39 (0.48)
Adolescents: CIB total	2.37 (0.37)	1.95 (0.67)	2.32 (0.27)	1.89 (0.61)
Child engagement	3.83 (0.66)	3.43 (0.75)	3.60 (0.48)	3.15 (0.71)
Child compliance	4.63 (0.37)	4.17 (0.75)	4.61 (0.31)	4.08 (0.74)
Child withdrawal	1.35 (0.28)	1.75 (0.63)	1.26 (0.24)	1.57 (0.52)
Dyad: CIB total	1.31 (0.54)	0.86 (0.84)	1.20 (0.83)	0.51 (1.08)
Dyadic reciprocity	4.10 (0.57)	3.65 (0.78)	4.16 (0.79)	3.30 (1.07)
Dyadic negative states	1.48 (0.57)	1.93 (0.95)	1.76 (0.91)	2.28 (1.15)

*HC-G* healthy control group, *BPD-G* borderline personality disorder group, *CIB* Coding Interactive Behavior

**Table 4:** Robust two-way mixed model analyses of variance: differences in maternal, adolescent and dyadic behavior depending on group and context

CIB scales	*F*	*df*	*p* value	*ges*
Main analyses				
Mothers: CIB total score				
Group	12.054	1, 39.3	0.001**^a^	.095
Context	2.593	1, 34.7	0.116^**a**^	.007
Group x context	0.006	1, 34.7	0.941^**a**^	.000
Adolescents: CIB total score				
Group	12.165	1, 28.2	0.001**^a^	0.152
Context	2.994	1, 32.1	0.093^a^	0.003
Group x context	0.009	1, 32.1	0.926^a^	0.000
Dyad: CIB total score				
Group	10.800	1, 29.3	0.003**^a^	0.106
Context	3.120	1, 32.4	0.087^**a**^	0.016
Group x context	1.887	1, 32.4	0.179^**a**^	0.004
Exploratory analyses				
Maternal sensitivity				
Group	10.231	1, 40.9	0.003**	0.076
Context	15.566	1, 40.7	< 0.001***	0.069
Group x context	0.239	1, 40.7	0.627	0.002
Maternal structuring				
Group	9.555	1, 31.1	0.004**	0.099
Context	1.483	1, 32.6	0.232	0.016
Group x context	0.185	1, 32.6	0.670	0.000
Maternal intrusiveness				
Group	1.028	1, 36.9	0.317	0.027
Context	0.382	1, 37.4	0.540	0.000
Group x context	1.286	1, 37.4	0.264	0.015
Child engagement				
Group	11.546	1, 35.7	0.002**	0.099
Context	12.868	1, 40.3	< 0.001***	0.037
Group x context	0.122	1, 40.3	0.728	0.000
Child compliance				
Group	12.009	1, 29.1	0.002**	0.156
Context	2.215	1, 31.9	0.146	0.002
Group x context	0.311	1, 31.9	0.581	0.001
Child withdrawal				
Group	8.728	1, 28	0.006**	0.137
Context	5.589	1, 30	0.025*	0.023
Group x context	0.549	1, 30	0.464	0.003
Dyadic reciprocity				
Group	16.503	1, 32.8	< 0.001***	0.144
Context	1.135	1, 37.8	0.293	0.007
Group x context	5.170	1, 37.8	0.029*	0.013
Dyadic negative states				
Group	6.115	1, 30.0	0.019*	0.066
Context	4.203	1, 30.8	0.049*	0.026
Group x context	0.413	1, 30.8	0.525	0.000

*n*_HC-G_ = 34, *n*_BPD-G_ = 37

*CIB* Coding Interactive Behavior

^a^Bonferroni-Holm corrected for multiple testing

^*^p < 0.05. **p < 0.01. ***p < 0.001

In exploratory analyses on subscale level, significant main effects for group were found for maternal sensitivity (*p* = 0.003) and maternal structuring (*p* = 0.004) but not for maternal intrusiveness (*p* = 0.317), all child scales [engagement: *p* = 0.002; compliance: *p* = 0.002; withdrawal: *p* = 0.006] and both dyadic scales [reciprocity: *p* < 0.001; negativity: *p* = 0.019]: in the BPD-G, mothers were less sensitive and structuring; adolescents behaved less engaged and compliant and more withdrawn. BPD-G dyads showed less reciprocity and more negativity in comparison to the HC-G. Context effects were significant for maternal sensitivity (*p* < 0.001), child engagement (*p* < 0.001), child withdrawal (*p* = 0.025) and dyadic negativity (*p* = 0.049): in both groups, mothers behaved less sensitive in the stress task compared to the positive interaction whilst adolescents displayed less engagement and less withdrawal. Dyadic negativity scores were higher in the stress task than in the positive interaction task. Context effects for maternal structuring and intrusiveness, child compliance and dyadic reciprocity were not significant. For dyadic reciprocity a significant group x context interaction (*p* = 0.029) was found: whilst HC-G dyads increased reciprocity under stress, BPD-G dyads decreased their reciprocal behavior. Details of all calculated ANOVAs are described in Table 4.

We next investigated, if SDQ total values were associated with CIB behavior. Exploratory analyses showed that SDQ was significantly negatively correlated with child and dyadic behavior during both interactions (*r* = *−* 0.24 to *r* = *− 0.36*) but not with maternal behavior. We then ran different regression analyses with group as a predictor and adolescent and dyadic behavior as outcome variables, controlling for SDQ total values. Whilst group still significantly predicted adolescent behavior during both tasks (positive interaction: *p* = 0.024, stress task: *p* = 0.044), the significant effect of group to dyadic behavior disappeared (positive interaction: *p* = 0.108, stress task: *p* = 0.141).

### The influence of childhood trauma on interactional behavior

Maternal CTQ total scores did not significantly correlate with any of the CIB scales and were therefore not considered in further analyses. Adolescent CTQ total scores were significantly negatively correlated with all CIB total scales (*r* = *−* 0.24 to *r* = *−* 0.40). Hierarchical linear regressions revealed that adolescent CTQ did not significantly explain any additional variance in CIB scores above and beyond group as a predictor (changes in *R*^*2*^: *p* = 0.078 to *p* = 0.425; see Table 5).

**Table 5:** Hierarchical linear regression models: adolescent early life maltreatment has no significant additional effect on maternal, adolescent or dyadic behavior

Variables	*B*	*SE*	*t*	*p*	*F*(*df*)	*p*	*R*^*2*^	∆*R*^*2*^	*p*∆*R*^*2*^
DV1: maternal behavior pos. IA									
Step 1: group	0.549	0.227	2.419	0.018*	5.851(1,71)	0.018*	0.076		
Step 2: group	0.403	0.291	1.387	0.170					
CTQ adolescent	− 0.117	0.146	− 0.802	0.425	3.233 (2,70)	0.045*	0.085	0.008	0.425
DV2: maternal behavior stress task									
Step 1^a^: group	0.665	0.222	3.001	0.004**	8.695 (1, 69)	0.004**	0.112		
Step 2: group	0.464	0.294	1.582	0.118					
CTQ adolescent	− 0.157	0.147	− 1.065	0.290	4.923(2,68)	0.010*	0.126	0.014	0.290
DV3: adolescent behavior pos. IA									
Step 1^a^: group	0.718	0.215	3.339	0.001**	10.661 (1, 71)	0.002**	0.131		
Step 2^a^: group	0.409	0.269	1.524	0.132					
CTQ adolescent	− 0.249	0.240	− 1.040	0.302	7.096 (2, 70)	0.002**	0.169	0.038	0.078
DV4: adolescent behavior stress task									
Step 1^a^: group	0.821	0.212	3.879	< 0.001***	14.182(1, 69)	< 0.001***	0.170		
Step 2^a^: group	0.522	0.251	2.082	0.041*					
CTQ adolescent	− 0.234	0.165	− 1.421	0.160	8.651(2, 68)	< 0.001***	0.203	0.032	0.101
DV5: dyadic behavior pos. IA									
Step 1^a^: group	0.606	0.221	2.743	0.008**	7.261(1, 71)	0.009**	0.093		
Step 2: group	0.377	0.286	1.318	0.192					
CTQ adolescent	− 0.184	0.144	− 1.280	0.205	4.483(2, 70)	0.015*	0.114	0.021	0.205
DV5: dyadic behavior stress task									
Step 1^a^: group	0.670	0.223	3.010	0.004**	8.856(1.69)	0.004**	0.114		
Step 2: group	0.479	0.293	1.631	0.108					
CTQ adolescent	− 0.150	0.147	− 1.018	0.312	4.949(2,68)	0.010*	0.127	0.013	0.312

positive interaction: *n*_HC-G_ = 35, *n*_BPD-G_ = 38, stress task: *n*_HC-G_ = 34, *n*_BPD-G_ = 37

*DV* dependent variable, *pos. IA* positive interaction, *CTQ* Childhood Trauma Questionnaire

^a^HC4 method for robust standard errors

^*^p < 0.05. **p < 0.01. ***p < 0.001

## Discussion

This paper is, to the best of our knowledge, the first to report on how current adolescent BPD traits are related to observed parental, adolescent and dyadic behavior during two different interactional contexts in a clinical sample. We also explored if adolescent and/or maternal ELM explain variance in behavioral outcomes in addition to BPD psychopathology.

Consistent with our main hypothesis, our clinical sample showed less quality in maternal, adolescent and dyadic behavior. On the subscale level, BPD-G displayed less maternal sensitivity and structuring, less child engagement and compliance and more child withdrawal as well as less dyadic reciprocity and more dyadic negative states in comparison to the HC-G. We therefore confirm earlier research reporting negative associations between BPD traits and quality of behavior in mother-adolescent interactions [34, 54] and extend these findings to an adolescent clinical sample in two different contexts.

Surprisingly, BPD-G mothers did not show significantly more intrusiveness than HC-G mothers. In adolescent community samples, increased maternal intrusiveness was found to be associated with BPD traits [34] and poorer adolescent psychological adaptation (a score based on the child’s externalizing, internalizing and depressive symptoms; [32]. Lyons‐Ruth & Yarger [62], however, suggested that maternal withdrawal might play a more crucial role in the development of adolescent BPD symptomatology than maternal intrusiveness. Children at risk for BPD might need more external regulation in order to manage their emotions and prevent dysfunctional emotion regulation like self-harming behavior (Lyons‐Ruth & Yarger, [62]). In the present study, maternal withdrawal would be represented by lower maternal sensitivity and structuring in BPD-G. Consistent with the studies reviewed by Lyons‐Ruth & Yarger [62], maternal sensitivity and structuring as protective factors might therefore be more relevant than the potential risk factor intrusiveness in the development of adolescent BPD.

Although maternal attachment style was previously shown to have an effect on parenting behavior in mother–child interactions [12] and there is also proof for a strong linkage between BPD and insecure attachment [1], we did not find maternal interpersonal sensitivity and attachment insecurity to be associated with behavior during either of the interactions. Future studies should explore if there are pathways through which maternal attachment styles and other maternal interpersonal relationship features contribute to the development of child or adolescent BPD traits.

We also wanted to know if adolescent general psychopathology has an effect on behavior, as most of our adolescent patients reported several comorbidities, a typical picture when assessing BPD samples [96]. Adolescent psychopathology in our sample was not related to maternal behavior. Whilst, when controlling for general psychopathology, group still significantly predicted adolescent behavior during both tasks, the significant effect of group on dyadic behavior disappeared. We can therefore not conclude that BPD traits alone predicted dyadic behavior, it might rather be a combination of BPD traits and general adolescent psychopathology. However, adolescent behavior during interactions might be reflecting interpersonal dysfunction (as a core symptom of the disorder) and therefore be a promising target for future research and interventional approaches.

As to context, we did not find significant main effects in our main analyses. On the subscale level, mothers were less sensitive during stress while adolescents showed less engagement and also less withdrawal which—on first sight—seems somewhat contradictory. These effects might be driven by different sample subgroups: some adolescents might disengage and some might become more active when being confronted with our stress task, independent of clinical status. Further analyses would be needed to explore possible factors that might help differentiate between adolescents using different emotion regulation strategies. On the dyadic level, dyads showed more negative states in the stress task compared to the fun day planning task. Although effect sizes of these context effects were only small to moderate and should be interpreted with caution, our results emphasize the need to investigate caregiver-child transactions in different contexts. As interpersonal stress in combination with a lack of emotion regulation skills (one of the core features of BPD) can be a trigger for impulsive and/or self-harming behavior (e.g. [44], a closer look at mother–child co-regulation under stress is warranted.

Another interesting result of our exploratory analyses was the fact that HC-G increased their reciprocal behavior during stress, i.e. their interaction became more fluent, compatible and interactive, whilst BPD-G decreased their reciprocal exchanges. Healthy dyads therefore seem to be able to rely on their abilities as a team to solve a potentially stressful situation by increasing their give-and-receive actions. BPD-G dyads, on the other hand, might not have trust in their partner: interactions between adolescents with BPD and their caregivers often provoke dysregulation and exacerbate dysfunctional behavior [40, 61]. Therefore, mothers might withdraw from the situation in expectation of a potential outburst of their child, and the child stops collaborating with their mother due to a lack of skills, overwhelming emotions they might feel and/or fear of an escalation of the situation. Research shows that adult BPD patients have problems with maintaining cooperation and repairing it when cooperation is ruptured [55]. Maybe these impairments could be addressed in early interventions focusing on dyadic behavior during interactions. However, due to small effect size this result has to be interpreted with care.

It is worthwhile to note that we did not find a greater amount of significant interactions, therefore, and contrary to our hypothesis, most group differences did not increase during stress. Fleck et al. [34] found BPD traits mostly associated with less behavioral quality during a stress paradigm but only few associations during a fun day planning task. Our results suggest that in a clinical sample, BPD-G dyads already struggle during tasks designed to elicit positive emotions (i.e. fun day planning), which might be reflecting the above-mentioned history of conflict-ridden interactions the BPD-G dyads have experienced in the past and a general lack of affiliative skills that are also needed in supposedly pleasant interactions.

Taken together, our results indicate that enhancing maternal sensitivity and reciprocal behavior during stress (which might increase maternal co-regulation when negative emotions are present) could be potential targets for BPD specific interventions in adolescence.

However, like most studies investigating parent–child interactions, we did not pursue a bidirectional and/or longitudinal approach, so we cannot determine how adolescents and mothers are influencing each other over time. Results of the Pittsburgh Girl study [53] with an at-risk community sample indicate that reciprocal effects of parental harsh punishment and adolescent poor self-control contribute to the development of adolescent BPD symptoms [43]. Longitudinally, maladaptive parental behavior caused increased adolescent BPD features which in turn led to worsening parental behavior [85]. Adolescent BPD symptoms at age 16 predicted greater parental BPD symptoms at age 17, whilst parental BPD symptoms did not influence adolescent BPD symptoms over time [52]. These results highlight the need to investigate both parent- and child-driven effects in reciprocal, longitudinal designs and during different developmental stages. During adolescence, for example, parental behavior might become less influential as achieving autonomy from parents and peer relationships become more important [52].

Our second research question was if and how maternal and/or adolescent ELM might influence behavior during mother-adolescent interactions. Maternal ELM was not correlated to behavioral outcomes during either of the interactions. Mothers of both groups showed similar levels of ELM, but BPD-G mothers reported higher levels of psychopathological symptoms than mothers of HC-G. Maternal psychopathology could be either a contributing factor for or a consequence of the offspring’s psychopathology. Literature shows that mothers of adolescents with BPD are more likely to have BPD or other affective disorders themselves [93]. Also﻿, resilience to trauma could be considered in this context: it has been previously discussed if maternal ELM alone or rather the combination with a mental disorder would negatively influence mother–child interactions [56, 65]. Therefore, although in our study mothers of both groups reported similar levels of ELM, the combination of maternal ELM and ensuing psychopathology might be contributing to the development of child BPD traits.

On the other hand, previous research shows that parents of mentally ill children report a decreased quality of life and more mental health problems than parents of healthy children [26]; this was also found for carers of individuals who suffer from BPD [6]. In exploratory analyses, maternal psychopathology did not correlate with any of the CIB scales and therefore does not seem to have an impact on behavioral outcomes in our sample. Importantly, maternal psychopathology did not reach clinically relevant cut-off values and values of both groups stand representative for the general population which might explain their negligible effect in our sample. Additionally, during adolescence, maternal influences might not have the same impact as during infancy or childhood [52].

Adolescent ELM was elevated in the BPD-G in comparison to the HC-G and correlated to all behavioral scales. It did not, however, explain more variance of behavior than clinical status alone which confirms previous findings about the strong link between ELM and the development of BPD (e.g. [48, 70]. It might be difficult to disentangle the effects of ELM and BPD traits as long as the adolescent is potentially still experiencing neglectful or traumatic familial circumstances. It can be assumed that ELM has already affected behavior but its effect is not (yet) distinguishable from BPD symptomatology. Future research should address this issue in longitudinal designs.

## Limitations

Although our study has several strengths (e.g. well characterized clinical sample, concurrent observation of maternal, adolescent and dyadic behavior), there are also limitations that should be discussed. As mentioned above, due to our cross-sectional design we could not investigate bidirectional transactions over time. In order to follow up on research questions that emerged during the discussion of our study results (e.g. the disentanglement of the influence of caregiver ELM, parental psychopathology and how this is related to parenting behavior and the occurrence of child psychopathology), bigger sample sizes would be needed.

Furthermore, only 50% of our clinical sample fulfilled full diagnostic criteria of BPD. Literature, however, suggests that even subthreshold BPD poses serious threats to mental health and psychosocial wellbeing and should therefore be treated in clinical settings and included in study designs [20, 50, 51, 87]. In our study, general adolescent psychopathology had an effect on behavior, especially on dyadic behavior. Future studies should include a second clinical sample (e.g. adolescents with Major depressive disorder) and always assess general psychopathology to improve the possibility to distinguish better between effects of pathological symptoms of any kind and of BPD traits.

Like most studies, we did not include fathers, although fathers have to be considered as well as sources of transgenerational mechanisms in the development of the disorder [75]. Our sample did also mainly comprise female BPD adolescents [boys: *n*_*BPD-G*_ = 6 (15.8%); *n*_*HC-G*_ = 7 (20%)] which did not allow us to pursue gender specific analyses. Our sample is highly educated which impedes comparisons to at-risk families that are typically affected by psychopathology. However, this also offers the chance to investigate BPD related familial mechanisms without the often-confounding factor of low socioeconomic status. Future studies should focus on clinical and more diverse samples with larger samples sizes, either applying longitudinal designs or minute-by-minute microcoding of behavior that would also allow the investigation of bidirectional transactions.

## Conclusion

The current study contributes to existing literature by demonstrating in a clinical sample a decreased quality of observed parental, adolescent and dyadic behavior during mother-adolescent interactions in two different contexts when adolescent BPD traits are present. Maternal and adolescent behavior during interactions seemed to be BPD-specific, whereas effects of BPD and general adolescent psychopathology overlapped regarding the prediction of dyadic behavior. Maternal ELM by itself did not seem to be associated with interactional behavior. Adolescent ELM contributed to negative behavior but did not have an effect additional to clinical status. Our data suggest a stronger focus on parent–child interactions (e.g. maternal sensitivity, adolescent behavior in general and dyadic reciprocity) in BPD specific therapies in order to improve interpersonal skills (especially in the context of stress) which could in turn enhance long-term treatment outcomes and psychosocial functioning for adolescent BPD patients.

## Data Availability

The study data are available from the corresponding author upon reasonable request.

## Abbreviations

BPD: Borderline personality disorder
BPD-G: Adolescent patients with borderline personality traits and their mothers
HC-G: Healthy control group
CIB: Coding interactive behavior
ELM: Early life maltreatment
SDQ: Strengths and difficulties questionnaire
CTQ: The childhood trauma questionnaire
VASQ: Vulnerable attachment style questionnaire
